# Metformin improves the weight reduction effect of mazindol in prediabetic obese Mexican subjects 

**DOI:** 10.5414/CP204180

**Published:** 2022-06-30

**Authors:** Federico Alberto Arguelles-Tello, Ashuin Kammar-García, Cristian Adolfo Trejo-Jasso, Juan Carlos Huerta-Cruz, Lina Marcela Barranco-Garduño, Héctor Isaac Rocha-González, Juan Gerardo Reyes-García

**Affiliations:** 1 Sección de Estudios de Posgrado e Investigación, Escuela Superior de Medicina, Instituto Politécnico Nacional,; 2 Dirección de Investigación, Instituto Nacional de Geriatría, and; 3 Unidad de Investigación en Farmacología, Instituto Nacional de Enfermedades Respiratorias, Ismael Cosio Villegas, Secretaría de Salud, México City, Mexico

**Keywords:** drug combination, glucose, mazindol, metformin, obesity, prediabetes

## Abstract

Abstract. Objective: Obesity is the strongest risk factor for type 2 diabetes (T2D). We aimed to explore 7% weight reduction rates of mazindol alone or combined with metformin in non-diabetic obese Mexican subjects who had additional risk factors for T2D. Materials and methods: In this randomized double-blind study, 137 participants received 1 mg mazindol (n = 65) alone or combined with 500 mg metformin (n = 72), twice a day, for 6 months. Results: Mazindol and mazindol-metformin were similarly effective. However, when subjects were subclassified into non-diabetics and prediabetics, according to glycated hemoglobin (HbA1c) – < 5.7% and 5.7 – 6.4%, respectively – and/or fasting plasma glucose (FPG) – < 100 mg/dL and 100 – 125 mg/dL, respectively –, differences were evident. Prediabetics in the mazindol-metformin group had a higher rate of 7% weight reduction (78.4%, n = 37) compared to prediabetics treated with mazindol (48.3%, n = 29). Furthermore, mazindol-metformin treatment induced significant reductions in fasting plasma insulin, HOMA-IR, and HbA1c in prediabetics compared to mazindol. No differences were found in any parameter between non-diabetics treated with mazindol (n = 36) and mazindol-metformin (n = 35). Conclusion: Our results highlight the effectiveness of mazindol-metformin to achieve higher rates of 7% weight reduction and to improve the glycemic profile in prediabetic obese subjects, which could be useful to prevent or delay T2D in these subjects.

**What is known about this subject**

- Mazindol is used as an adjuvant drug to reduce body weight in obese patients.
- Metformin has been recommended to prevent or delay the onset of diabetes in patients with risk factors for diabetes.
- Obesity is associated with an increased incidence of type 2 diabetes.

**What this study adds**

- Metformin improves the 7% weight reduction rate of mazindol in prediabetic patients.
- Metformin-mazindol combination prompted significant improvements in glycemic parameters of prediabetic subjects.
- Metformin-mazindol treatment could be useful to prevent or delay type 2 diabetes mellitus in prediabetic obese subjects.

## Introduction 

Diabetes mellitus is a serious chronic disease associated with high morbidity – including ischemic heart disease, stroke, kidney disease, peripheral vascular disease, vision loss, among others – that can increase the overall risk of premature death [1, 2]. The prevalence of diabetes among adults over 18 years has been estimated at 8.5 and 9% worldwide and in the Mexican population, respectively [2, 3]. The high morbidity, reduced life expectancy, and costs associated with diabetes make this an important public health concern. Type 2 diabetes mellitus (T2D) is the most common form of this disease [1, 2]. Insulin resistance and alterations in relative insulin secretion have been implied as the main pathophysiological causes of T2D [4]. Obesity is considered the strongest etiological risk factor for T2D, and it has been estimated that this condition contributes to 55 – 90% of cases [2, 5]. Risk factors for T2D include increasing age, ethnicity, having a first-degree relative with diabetes, high blood pressure, sedentarism, impaired fasting blood glucose, and high lipid blood levels [6]. Dietary factors like consumption of red meats and sugar-sweetened beverages, as well as reduced intake of fruit and vegetables are also important risk factors [7, 8, 9, 10]. 

A healthy lifestyle including healthy diet, physical activity, and body weight reduction may prevent T2D in subjects who have glucose intolerance – with or without impaired fasting plasma glucose [2, 9]. Additionally, pharmacological interventions can prevent or delay T2D [11]. In this regard, the Diabetes Prevention Program (DPP) demonstrated a significant reduction in the incidence of T2D after 2.8 years in subjects managed with an intensive lifestyle intervention – diet and exercise – or metformin plus standard diet and exercise [12]. One of the main goals of the DPP was to achieve and maintain a 7% weight loss for 24 weeks; however, only 50% of participants in the lifestyle intervention group achieved this objective. Furthermore, mean weight losses were only 2.1 and 5.6 kg in the metformin and lifestyle-intervention groups, respectively [12]. Monotherapy for weight loss has only shown modest efficacy to date, which is often not sustained for long periods. Therefore, combinations of drugs are becoming more common and accepted strategies to increase weight loss efficacy and improve other metabolic parameters while minimizing adverse events. Thus, the combination of antiobesity drugs with metformin could be useful to loose weight and prevent T2D in a timely manner. 

Mazindol is a sympathomimetic amine still marketed as a weight loss medication in countries like Mexico, Brazil, Japan, among others. It induces weight loss through direct suppression of appetite centers located in the lateral hypothalamus by stimulating the release of catecholamines [13]. In addition, it has been suggested that mazindol inhibits glucose absorption [14, 15]. Correspondingly, metformin is a biguanide that induces weight loss by inhibiting the activity of AMP-activated protein kinase in hypothalamic neurons and by improving sensitivity to insulin [16]. Thus, this study aimed to explore the 7% weight reduction rate and metabolic effects of mazindol as a single therapy or a fixed dose of mazindol-metformin in non-diabetic obese Mexican subjects with additional risk factors for T2D. 

## Materials and methods 

### Study design 

A double-blinded, randomized clinical trial was performed, with 137 subjects. All of them had at least one additional risk factor – besides obesity and race – for T2D: age (> 45 years), first-degree relative with diabetes, hypertension, high triglyceride blood levels, physical inactivity, or prediabetes – defined as two consecutive measures of glycated hemoglobin (HbA1c) of 5.7 – 6.4%, and/or fasting plasma glucose (FPG) of 100 – 125 mg/dL. Exclusion criteria were hypersensitivity to sympathomimetic or biguanide drugs, use of other antiobesity drugs, T2D, uncontrolled arterial blood pressure, other diseases – pulmonary, renal, hepatic, endocrine, cardiac, or psychiatric disorders –, history of substance abuse, and pregnancy. The study was carried out between September 27, 2016 and October 2, 2017 in the Asociación Mexicana para la Investigación Clínica, A.C. (AMIC). 

This study is in line with the recommendations of the latest version of the World Medical Association Declaration of Helsinki – Ethical Principles for Medical Research Involving Human Subjects [17]. This study was also approved by the Institutional Research and Ethics Committees of the General Hospital of Hidalgo, and the Mexican Federal Commission for Protection against Health Risks (protocol number 163300CT190261/2016). Participants were able to read the research protocol and provided their written informed consent to participate in the study. After obtaining written informed consent, subjects were randomly allocated to one of two groups to receive either 1 capsule of mazindol (1 mg) or mazindol-metformin (1 mg/500 mg), twice a day (BID), for 6 months. All capsules were provided by Productos Medix, S.A. de C.V. (Mexico City, Mexico). 

Subjects were advised to follow a hypocaloric diet – 600 kcal deficit – and to perform physical activity for 30 minutes per day. The variables body weight (BW) – used to determine 7% BW loss – , body mass index (BMI), waist circumference (WC), systolic blood pressure (SBP), and diastolic blood pressure (DBP) were evaluated at 0 – 6 month visits, whereas fasting serum triglycerides (TGL), fasting plasma glucose (FPG), fasting plasma insulin (FPI), and glycated hemoglobin (HbA1c) were measured at months 0, 3, and 6. The homeostasis model assessment-estimated insulin resistance (HOMA-IR) was calculated from FPG and FPI values [18]. 

Safety was evaluated every month through directed anamnesis and physical examination, and by reviewing the patient’s diary. At every visit, patients were required to empty their bladder upon arrival and dress with a hospital gown to determine nude BW with a calibrated scale. Height was assessed with the patient standing with the heels together, and the buttocks, shoulders, and head in contact with the stadiometer. WC was measured by placing a flexible metric tape at the level of the umbilicus without making pressure; subjects with BMI > 45 were not included. Measurements of SBP and DBP were obtained using an electronic sphygmomanometer. FPG, FPI, HbA1c, and TGL were determined by blood chemistry tests. 

### Data analysis 

Data from subjects who completed the study and had at least 80% drug adherence were analyzed according to treatment and prediabetes status. Age, BW, BMI, WC, SBP, DBP, FPG, FPI, HbA1c, TGL, and HOMA-IR are presented as mean ± standard deviation (SD), whereas gender, risk factors for T2D, 7% BW loss, and adverse events are presented as frequencies and percentages in tables or graphs. Statistical differences were obtained by Student’s t-test, χ^2^-test, or repeated measures ANOVA models followed by Bonferroni’s test. The statistical assumptions of ANOVA models were corroborated through residual analysis. In all cases, differences were considered statistically significant with a p ≤ 0.05. Statistical analyses were performed using the SPSS v.22.0 software, and figures were created in GraphPad Prism 6. 

## Results 

### Demographic and baseline data 

There were no significant differences in baseline characteristics between both groups. Most subjects were young women with class I obesity and WC above the upper reference limit. Mean values of SBP, DPB, TGL, FPG, FPI, and HbA1c were very close to reference limits. In addition, the mean HOMA-IR was slightly higher in the mazindol-metformin group compared with the mazindol group. Drug adherence (~ 90%) and diet adherence (~ 70%) were similar in both groups (Table 1). 

### Risk factors for diabetes 

The mean counts of risk factors for diabetes were 4.7 ± 0.7 and 4.5 ± 0.9 in the mazindol and mazindol-metformin groups, respectively. After ethnicity and obesity, sedentarism was the most frequent risk factor, being present in more than 80% of subjects in both groups. A significantly higher proportion of subjects with a history of first-degree relatives with diabetes was present in the mazindol group (p < 0.05). Approximately 30% of subjects were > 45 years in both groups. Hypertension and hypertriglyceridemia were present in a low percentage of subjects in both groups. Nearly 50% of subjects had increased FPG and/or HbA1c levels in both groups. Except for having a first-degree relative with diabetes, there were not statistically significant differences between the groups (Table 2). 

### Efficacy of mazindol and mazindol-metformin 

Table 3 shows the mean changes in variables under investigation for subjects in the mazindol-only and mazindol-metformin groups. Treatment with mazindol alone or in combination with metformin significantly reduced mean baseline BW by months 3 (–6.4 and –7.3 kg, respectively) and 6 (–8.6 and –9.9 kg, respectively). BMI and WC were also proportionally significantly lower. A greater but non-statistically different proportion of subjects in the mazindol-metformin achieved a ˃ 7% BW reduction compared with the mazindol-only group at months 3 (68.1 vs. 52.3%) and 6 (77.8 vs. 69.2%). In addition, the Pearson’s coefficient of correlation between % BW reduction and % diet adherence showed a weak correlation (r = 0.1847). 

SBP but not DBP tended to be lower in both groups at months 3 and 6, the latter reaching statistical significance. With respect to biochemical determinations, both mazindol and mazindol-metformin produced similar sustained reductions in TGL (approximately –25 to –30 mg/dL) at months 3 and 6, which were statistically significantly different from their baseline values, but not between treatment groups. 

Mean baseline FPG, FPI, and HbA1c values were within reference limits. Mazindol and mazindol-metformin induced slight but significant FPG reductions, which were more pronounced at the 3^rd^ month compared with the 6^th^ month. A significant FPI reduction was observed in mazindol-metformin, but not in the mazindol group at months 3 and 6. None of these treatments produced relevant changes in HbA1c. Mean HOMA-IR was significantly reduced by months 3 and 6 in the mazindol-metformin group, whereas HOMA-IR values in the mazindol-only in the mazindol-only group also had a tendency towards reduction which was only statistically significantly different at the 3^rd^ month. 

### Subclassification of subjects as non-prediabetic and prediabetic 

**Figure 1:**
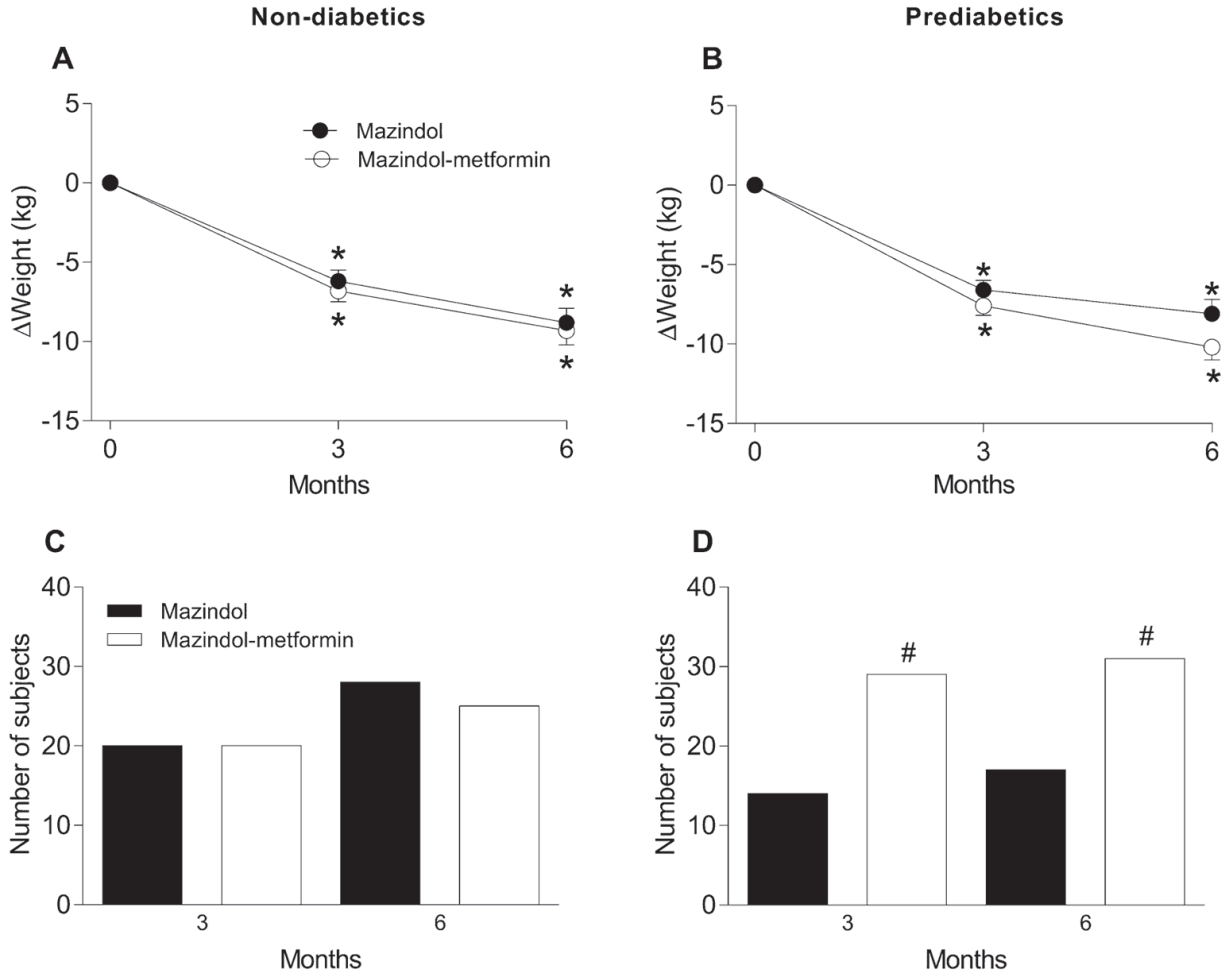
Time courses showing the effect of mazindol alone and mazindol-metformin in non-prediabetic (29 and 37 subjects, respectively) and prediabetic (36 and 35, respectively) subjects on body weight reduction (A, B) and achievement of the ˃ 7% weight reduction goal (C, D). *Significantly different with respect to baseline values, but not between groups (p < 0.05), as determined by repeated measures ANOVA followed by post-hoc Bonferroni’s test; ^#^significantly different compared with the mazindol group by χ^2^-test.

Subjects were subclassified as non-prediabetic (NPD) or prediabetic (PD) according to HbA1c (< 5.7% and 5.7 – 6.4%, respectively) and/or FPG (< 100 and 100 – 125 mg/dL, respectively). NPD subjects treated with mazindol (NPD-M; 89.6 ± 16.1 kg) or mazindol-metformin (NPD-MM; 92.8 ± 16.6 kg) had a higher mean baseline weight than PD subjects treated with mazindol (PD-M; 84.9 ± 11.7 kg) or mazindol-metformin (PD-MM; 84.4 ± 12.9 kg). The weight reduction pattern was similar in the NPD subgroups by months 3 (NPD-M = –6.2 kg vs. NPD-MM = –6.8 kg) and 6 (NPD-M = –8.8 kg vs. NPD-MM= –9.3 kg) (Figure 1A). Weight loss in the PD subgroups had a tendency to be higher in PD-MM than PD-M by the 3^rd^ month (PD-M = –6.6 kg vs. PD-MM = –7.6 kg), on the border of significance (p = 0.06) by the 6^th^ month (PD-M = –8.1 kg vs. PD-MM = –10.2 kg) (Figure 1B). There were no significant differences in the percentage of NPD subjects achieving ˃ 7% weight reduction in the NPD-M and NPD-MM subgroups at months 3 (55.6 vs. 57.1%) and 6 (77.8 vs. 71.4%) (Figure 1C). Conversely, a significantly higher percentage of PD subjects in the PD-MM subgroup had a ˃7% weight reduction compared with the PD-M subgroup at months 3 (78.4 vs. 48.3%) and 6 (83.8 vs. 58.6%) (Figure 1D). In all four subgroups, there were similar significant reductions in BMI, WC, and SBP, whereas DPB was unchanged. 

**Figure 2:**
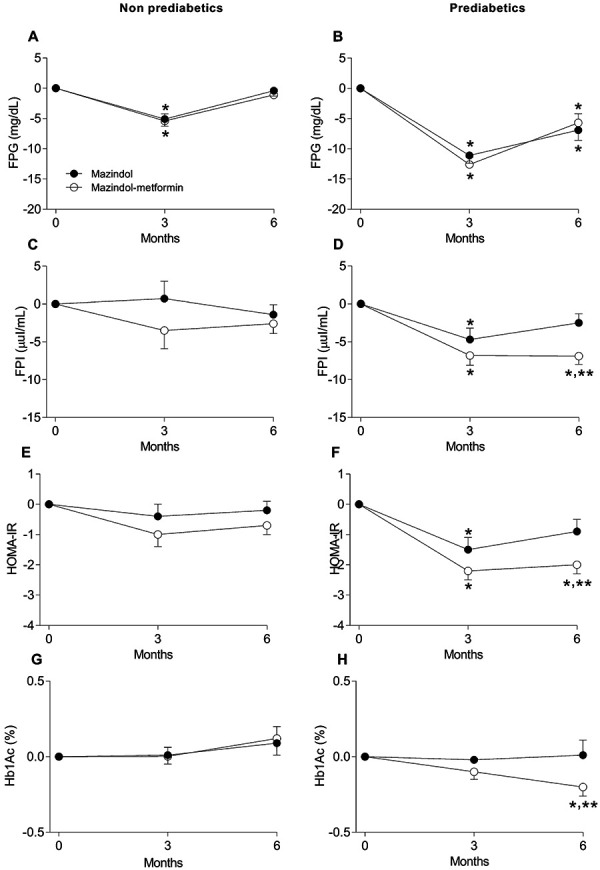
Time courses showing the effect of mazindol alone and mazindol-metformin in non-prediabetic (29 and 37 subjects, respectively) and prediabetic (36 and 35, respectively) subjects on fasting plasma glucose (FPG) reductions (A, B), fasting plasma insulin (FPI) (C, D), homeostatic model assessment for insulin resistance (HOMA-IR) (E, F), or glycated hemoglobin (HbA1c; G, H). *Significantly different with respect to baseline values; **significantly different between subgroups (p ≤ 0.05), as determined by repeated measures ANOVA followed by post-hoc Bonferroni´s test.

For NPD, laboratory determinations of FBP (Figure 2A), FPI (Figure 2C), HOMA-IR (Figure 2E), and HbA1c (Figure 2G) showed only discrete changes in the NPD-M and NPD-MM subgroups. Only significant reductions in FPG (Figure 2A) were observed in both subgroups at month 3. 

Conversely, PD-MM subjects had significantly greater reductions of FPI (Figure 2D), HOMA-IR (Figure 2F), and HbA1c (Figure 2H) compared with PD-M patients at month 6. However, FPG (Figure 2B), FPI (Figure 2D), and HbA1c (Figure 2H) were within range or only slightly above the reference interval in both subgroups. Both PD subgroups showed reductions in mean FPG, which was sustained up to the reference range by the 6-month visit (Figure 2B). The PD-MM subgroup also had sustained and significantly lower 3-month and 6-month FPI (Figure 2D) and HOMA-IR (Figure 2F), as well as significantly improved 6-month HbA1c (Figure 2H), whereas the PD-M subgroup only had significant reductions in 3-month FPG (Figure 2B), FPI (Figure 2D), and HOMA-IR (Figure 2F), but not in HbA1c (Figure 2H). 

Regarding TGL, all mazindol and mazindol-metformin subgroups had similar 3- and 6-month sustained and significant reductions of approximately –25  to –40 mg/dL, with no statistically significant differences between them. 

### Safety of treatments 

137 subjects completed the study and met at least 80% treatment compliance. The main reason for non-compliance was subject self-withdrawal. Adverse events reported with a frequency greater than 10% in the mazindol-metformin and mazindol groups, respectively, were nausea (22 vs. 10%), constipation (15 vs. 13%), dry mouth (10 vs. 16%), and headache (11 vs. 24%); diarrhea was less common (5 vs. 4%). Hypoglycemia was not reported. Adverse events were mostly mild, and only 2 serious adverse events in the mazindol group were reported (radius and ulna fractures, and cholelithiasis), none of which were classified as being related to the drug. Only 2 patients abandoned the study due to adverse events (erectile dysfunction and palpitations), both from the mazindol-metformin group. 

## Discussion 

This study aimed to assess the short-term efficacy and safety of mazindol alone (1 mg, BID), or combined with metformin (1 mg plus 500 mg, BID) in obese Mexican subjects at high risk of T2D, mainly in terms of weight reduction and improvement of glycemic profile. We found that daily oral administration of mazindol and mazindol-metformin for 6 months led to similar reductions in anthropometric variables such as BW, BMI, and WC. Moreover, 69.2 and 77.8% of subjects in the mazindol and mazindol-metformin groups, respectively, achieved the 7% weight reduction target by the end of the study. Both treatments led to similar reductions in FPG and TGL, but only mazindol-metformin was related to sustained reductions in FPI and HOMA-IR. In addition, HbA1c and blood pressure remained mostly unchanged in both treatment groups. Regarding the efficacy of mazindol on weight reduction, our results are in agreement with 2- to 16-week studies [19, 20, 21, 22]. Unlike our trial, other studies also found improvements in insulin sensitivity and other glycemic parameters alongside BW reduction [13, 14]. These differences could be because our patients were mostly in the class 1 obesity category, whereas other studies have included severely obese subjects – who could have greater insulin resistance – , under very low-calorie diets. 

We found that the addition of metformin to mazindol likely improved the glycemic profile of subjects in a higher degree than mazindol alone. Nonetheless, there were no significant differences in BW reduction nor the number of patients who achieved the 7% weight reduction goal between both treatment groups. This result disagrees with a previous study reporting a weight reduction of –5.8 ± 7.0 kg after 6 months of treatment with metformin in 154 non-diabetic subjects. Such difference could be explained by the fact that we used daily doses of 1,000 mg metformin, compared with the daily 2,500 mg used previously [23]. Interestingly, the proportion of subjects that achieved a ˃ 7% BW reduction (~ 70%) was close to the percentage of diet adherence (~ 70%). However, the Pearson’s coefficient of determination between % BW reduction and % diet adherence was 0.0341, indicating that the % BW reduction can be attributed to pharmacological treatment rather than diet adherence. Altogether, our results apparently point towards a similar effectiveness of mazindol alone or combined with metformin to achieve a 7% weight reduction in obese subjects at high risk of T2D, with slightly better improvements in the glycemic profile by combining mazindol with metformin. 

In our study, the subclassification of subjects in NPD or PD according to HbA1c (< 5.7% and 5.7 – 6.4%, respectively) and/or FPG (< 100 mg/dL and 100 – 125 mg/dL, respectively) levels showed that both NPD and PD under either treatment scheme had similar weight loss time-courses, with borderline significance to greater reductions in the PD-MM subgroup (10.2 kg at month 6). Furthermore, a significantly higher number of PD-MM subjects reached the 7% BW loss target after 6 months compared with PD-M patients (~ 80% and ~ 60%, respectively). Interestingly, 78.4% of PD-MM subjects achieved the weight loss target after 3 months of treatment. 

On a different note, NPD-M and NPD-MM subgroups only showed transient reductions in FPG at 3 months with respect to their baseline values, while the PD-M subgroup displayed significant reductions in mean FPG, as well as transient improvements in FPI and HOMA-IR; no changes in HbA1c were observed. Contrarily, the PD-MM subgroup exhibited sustained and significant reductions in FPG, FPI, HOMA-IR, and HbA1c. However, it is important to observe that the PD-MM subgroup had significantly higher reductions in FPI, HOMA-IR, and HbA1c, compared with the PD-M subgroup. Our findings in NPD subjects are consistent with those reported in a previous open-label study assessing the combination of liraglutide – a glucagon-like peptide-1 analog (1.2 mg QID) – plus metformin (1,000 mg BID) for 3 months in 36 women with polycystic ovary syndrome, of whom only 33% had impaired glucose tolerance. In this study, a non-significant tendency towards improved weight loss was observed in those treated with liraglutide plus metformin compared with liraglutide alone, while more subjects in the liraglutide plus metformin group reached a weight reduction ≥ 5% and had improved HOMA-IR scores. FPG and FPI did not consistently improve in any of the treatment arms. In addition, weight reduction was achieved in a shorter period than usual with liraglutide alone [24]. 

Although there are no studies that could be directly compared to our findings in PD subjects, the observed improvements in weight reduction and glycemic profile in PD-MM could be explained by the potential association between weight loss and improved glycemic profiles with mazindol and metformin. In this sense, metformin is an insulin-sensitizing agent that improves blood glucose levels by decreasing hepatic gluconeogenesis and suppressing insulin production [25]. Emerging evidence suggests that metformin-associated weight loss is attributable to anorectic effects since it inhibits the activity of AMP-dependent protein kinases in the central nervous system, resulting in a decrease in the expression of the orexigenic neuropeptide Y (NPY), and an increase in the anorectic peptide pro-opiomelanocortin (POMC) in the hypothalamus [16]. Furthermore, metformin increases leptin expression and increases glucagon-like peptide 1 (GLP-1) levels through enzymatic inhibition of dipeptidyl peptidase IV [16, 26, 27]. In addition, it modulates peptide-releasing gastrin and muscarinic signaling pathways, as well as the microbiome [25, 28]. Complementarily, mazindol induces weight loss through direct suppression of appetite centers located in the lateral hypothalamus by stimulating the release of catecholamines [13]. Furthermore, mazindol inhibits glucose intestinal absorption [22] and decreases the activity of glucose-sensitive neurons located in the lateral hypothalamus, resulting in decreased gastric acid secretion, reduced appetite, and lower glucose levels [29]. 

In our study, mazindol and mazindol-metformin were well tolerated, with similar adverse events to those reported in other studies [30, 31]. The most frequent adverse events were gastrointestinal in both treatment groups. There were few cases of diarrhea – an adverse event frequently reported with metformin treatment – which could have been diminished by the constipating effect of mazindol. 

Limitations of our study include a small sample size mainly conformed by Mexican women younger than 45 years – which could limit extrapolation of our results to other populations –, the non-inclusion of a metformin-only treatment arm, and the variability in some baseline parameters between subgroups. Despite these limitations, our results point towards an additive effect of metformin plus mazindol for the treatment of obesity in prediabetic patients but not in non-prediabetic subjects. Their effects on BW reduction and improvement of insulin resistance could prevent or delay the onset of T2D. 

## Conclusion 

Our results highlight the potential usefulness of the short-term weight loss agent mazindol alongside metformin to achieve higher rates of 7% weight reduction in comparison to mazindol alone in prediabetic obese subjects. In addition, mazindol-metformin combination improves the glycemic profile in prediabetic obese subjects, but not in non-prediabetic obese subjects. This treatment could be useful to prevent or delay T2DM in this group of subjects. 

## Acknowledgment 

This work is part of the PhD dissertation of Federico Alberto Arguelles Tello. The authors kindly thank Cecilia Fernandez del Valle-Laisequilla from Productos Medix, S.A. de C.V. for her support throughout this study. 

## Authors’ contributions 

JGR-G and HIR-G conceptualized the idea and designed the study; FAA-T, AK-G, CAT-J, LMB-G, and J-RS performed the acquisition of the data; AK-G, CAT-J, and JCH-C analyzed the data; and JGR-G, HIR-G, AK-G, and CAT-J interpreted the data for the work. All authors participated in the review, correction, and improvement of the manuscript. All of them approved the final version and agree to be accountable for all aspects of the work in ensuring that questions related to the accuracy or integrity of any part of the work are appropriately investigated and resolved. 

## Funding 

This work was partially supported by Conacyt grant 213481. 

## Conflict of interest 

We have no conflict of interest to disclose. 

**Table 1.** Baseline characteristics and treatment adherence of patients in the study population.

Characteristic	Reference value	Mazindol (n = 65)	Mazindol-metformin (n = 72)
Gender, n (%)			
Female	–	55 (84.6)	63 (87.5)
Male	–	10 (15.4)	9 (12.5)
Age, years mean (SD)	–	39.8 (10.1)	39.4 (9.6)
Weight, kg mean (SD)	–	87.5 (14.4)	88.50 (15.3)
BMI, kg/m^2^ mean (SD)	< 30	34.7 (3.8)	34.7 (3.8)
Waist circumference, cm mean (SD)			
Female	≤ 88	106.5 (11.3)	107.8 (10.5)
Male	≤ 102	114.2 (9.7)	117.8 (10.5)
SBP, mmHg mean (SD)	< 120	119.0 (13.1)	119.6 (10.8)
DBP, mmHg mean (SD)	< 80	77.1 (7.5)	79.1 (7.2)
TGL, mg/dL mean (SD)	< 150	148.2 (57.5)	152.3 (69.0)
FPG, mg/dL mean (SD)	75 – < 99	97.5 (6.9)	98.9 (7.6)
FPI, μUI/mL mean (SD)	0 – < 25	19.7 (8.8)	22.1 (12.7)
HbA1c, % mean (SD)	4.5 – < 5.7	5.5 (0.4)	5.6 (0.3)
HOMA-IR, mean (SD)	–	4.7 (2.1)	5.4 (3.3)
Drug adherence, n (%)	–	61 (93.8)	67 (93.1)
Diet adherence, n (%)	–	46 (70.7)	50 (69.4)
Exercise adherence, n (%)	–	38 (58.4)	44 (61.1)

Data are expressed as mean (SD) or as number of patients (%). There is no difference by Student’s t-test or χ^2^-test. BMI = body mass index; DBP = diastolic blood pressure; FPG = fasting plasma glucose; FPI = fasting plasma insulin; TGL = fasting serum triglycerides; HbA1c = glycated hemoglobin; HOMA-IR = homeostatic model assessment for insulin resistance; SBP = systolic blood pressure.

**Table 2.** Risk factors for diabetes according to treatment groups.

Risk factor	Mazindol (N = 65)	Mazindol-metformin (N = 72)
Number of risk factors, mean (SD)	4.7 ± 0.7	4.5 ± 0.9
Obesity, n (%)	65 (100%)	72 (100%)
Latin race, n (%)	65 (100%)	72 (100%)
First-degree relative with diabetes, n (%)	36 (55.3%)	28 (38.8%)*
History of hypertension, n (%)	2 (3.0%)	2 (2.7%)
Hypertriglyceridemia, n (%)	3 (4.6%)	6 (8.3%)
Sedentarism, n (%)	55 (84.6%)	60 (83.3%)
Age > 45 years, n (%)	23 (35.3%)	20 (27.7%)
Prediabetes according to HbA1c and/or glycemia, n (%)	29 (44.6%)	37 (51.3%)

Data are expressed as mean (SD) or as number of patients (%). *Significantly different (p < 0.05), as determined by χ^2^-test. HbA1c = glycosylated hemoglobin.

**Table 3.** Treatment with mazindol or mazindol-metformin in subjects with risk factors to develop type 2 diabetes.

Characteristic	Baseline		Month 3		Month 6	
	M (n = 65)	MM (n = 72)	M (n = 65)	MM (n = 72)	M (n = 65)	MM (n = 72)
Weight, kg mean (SD)	87.5 (14.4)	88.5 (15.3)	81.1 (13.9)*	81.2 (14.7)*	78.9 (13.8)*	78.6 (14.9)*
BMI, kg/m^2^ mean (SD)	34.7 (3.8)	34.7 (3.9)	32.1 (3.8)*	31.8 (3.9)*	31.4 (4.1)*	30.8 (4.1)*
WC, cm mean (SD)	106.5 (11.3)	107.8 (10.5)	97.5 (9.9)*	97.4 (10.3)*	93.3 (10.1)*	92.9 (9.9)*
Weight loss ˃ 7%, n (%)	–	–	34 (52.3)	49 (68.1)	45 (69.2)	56 (77.8)
SBP, mmHg mean (SD)	119.0 (13.1)	119.6 (10.8)	115.7 (11.9)	115.4 (12.3)	112.5 (11.8)*	114.5 (11.7)*
DBP, mmHg mean (SD)	77.1 (7.5)	79.1 (7.2)	80.0 (6.8)	80.3 (6.8)	76.6 (7.5)	78.5 (7.4)
TGL, mg/dL mean (SD)	148.2 (57.5)	152.3 (69.0)	121.7 (54.6)*	120.7 (63.5)*	117.2 (53.3)*	124.2 (74.0)*
FPG, mg/dL mean (SD)	97.5 (6.9)	98.9 (7.6)	89.9 (7.6)*	89.8 (8.0)*	94.4 (6.8)*	95.5 (7.6)*
FPI, μUI/mL mean (SD)	19.7 (8.8)	22.1 (12.7)	18.0 (15.5)	16.9 (10.7)*	17.9 (10.3)	17.2 (9.9)*
HOMA-IR, mean (SD)	4.7 (2.1)	5.4 (3.3)	3.8 (2.8)*	3.8 (2.7)*	4.2 (2.5)	4.1 (2.4)*
HbA1c, % mean (SD)	5.5 (0.4)	5.6 (0.3)	5.5 (0.4)	5.5 (0.3)	5.5 (0.5)	5.5 (0.3)

Data are expressed as mean (SD) or the number of subjects (%). *Significantly different (p < 0.05) in relation to baseline values, as determined by repeated measures ANOVA followed by post-hoc Bonferroni’s test. There is no difference among groups. BMI = body mass index; DBP = diastolic blood pressure; FPG = fasting plasma glucose; FPI = fasting plasma insulin; TGL = fasting serum triglycerides; HbA1c = glycosylated hemoglobin; HOMA-IR = homeostatic model assessment for insulin resistance; M = mazindol treatment; MM = mazindol-metformin treatment; SBP = systolic blood pressure; WC = waist circumference.
